# Research progress of piezoelectric materials in protecting oral health and treating oral diseases: a mini-review

**DOI:** 10.3389/fbioe.2024.1473126

**Published:** 2024-09-13

**Authors:** Tingyu Yang, Rina Sa, Furong Wang, Chen Chen, Lanbing Zheng

**Affiliations:** ^1^ Scientific Research Department, Inner Mongolia Fourth Hospital (Chest Hospital), Hohhot, China; ^2^ The Department of Infectious Diseases, Inner Mongolia Fourth Hospital (Chest Hospital), Hohhot, China

**Keywords:** piezoelectric material, teeth whitening, oral disease treatment, treatment mechanism, ROS, electrical stimulation

## Abstract

Piezoelectric materials, as a class of materials capable of generating electrical charges under mechanical vibration, have special piezoelectric effects and have been widely applied in various disease treatment fields. People generate vibrations in the oral cavity during daily activities such as brushing teeth, using electric toothbrushes, chewing, and speaking. These natural vibrations (or external ultrasound) provide ideal conditions for activating piezoelectric materials, leading to their high potential applications in protecting oral health and treating oral diseases. Based on this, this review reports on the research progress and trends of piezoelectric materials in the protection of oral health and the treatment of oral diseases in the past 5 years, and discusses its treatment mechanism, challenges and shortcomings, aiming to provide theoretical basis and new ideas for the future application of piezoelectric materials in the field of oral cavity. Finally, a brief outlook is provided, suggesting that the potential of piezoelectric materials may enable them to quickly move towards real clinical applications.

## 1 Introduction

Oral health is significant for people’s daily physiological activities (WHO, 2022). More and more people are suffering from oral health problems and oral diseases due to poor personal dietary and hygiene habits (Baker et al., 2024). Oral diseases have become a public health issue, seriously affecting people’s physiological and health functions (WHO, 2022). In particular, some oral diseases may also lead to systemic disease risks (Botelho et al., 2022), such as cardiovascular disease (Tonelli et al., 2023) and diabetes (Li Y. et al., 2024). In addition, oral infections and the growth of bacteria in the mouth may also be transmitted through saliva, increasing the risk of infectious diseases (Jiang et al., 2021). Therefore, how to maintain oral health and achieve efficient treatment of oral diseases has become an urgent challenge in the global public health field.

Currently, many dental materials have been applied clinically to maintain oral health and treat oral diseases (Jia et al., 2024). However, due to the complex oral environment and the presence of a large number of oral microorganisms, many dental materials fail to perform as intended (Jia et al., 2024). For example, dental resin materials are easily degraded by enzymes from saliva and bacteria, as well as acid produced by bacteria (Montoya et al., 2021a). To address the aforementioned oral issues, an increasing number of functional biomaterials are applied in the treatment of oral diseases (Ding et al., 2024; Wei et al., 2024a; Wang Y. et al., 2024). Among them, piezoelectric materials, due to their good biocompatibility and biosafety, as well as their unique properties compared to other biomaterials, can generate charge separation under mechanical stress or ultrasound, which can stimulate the production of reactive oxygen species in the environment and other special functions (electrical stimulation effect, improvement of cell function, anti-inflammatory effect, acceleration of tissue repair and regeneration, etc.), have become a new emerging material with strong application potential in the treatment of oral diseases (Wang et al., 2020; Roldan et al., 2023; Liu X. et al., 2024; Chernova et al., 2024).

In the past 5 years, a large number of review articles have been published on piezoelectric materials as a type of biomaterial with special functions, detailing their preparation process, mechanism, and applications in biomedical fields such as tumor treatment and tissue repair and regeneration (Wang J. et al., 2024; Dai et al., 2024; Zheng et al., 2024). Wang et al. review the basic principles and applications of piezoelectric materials in tumor therapy (Wang Y. et al., 2023). Nain et al. discuss the role of piezoelectric materials in tissue remodeling (Nain et al., 2024). However, to our knowledge, there is currently no comprehensive systematic review solely dedicated to the application of piezoelectric materials in the field of oral. Existing reviews mainly focus on the applications of piezoelectric materials in other biomedical fields, with only a small portion briefly mentioning their potential application of oral therapy (Nain et al., 2024; Chen S. et al., 2023; Wang L. et al., 2023). Here, this review will not specifically discuss aspects such as the preparation methods of piezoelectric materials that have already been extensively covered in other reviews.

**FIGURE 1:**
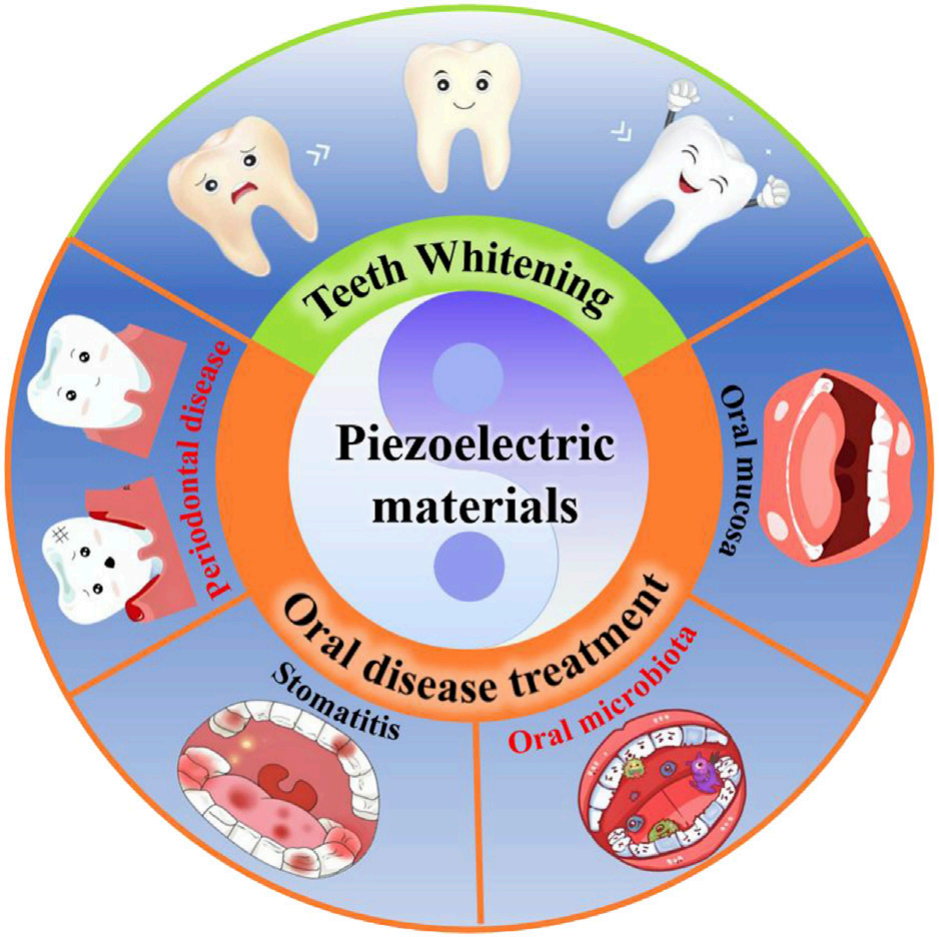
Schematic diagram of piezoelectric materials in protecting oral health and treating oral diseases.

In this review, we aim to focus on the latest research progress of emerging piezoelectric materials in protecting oral health and treating oral diseases. Firstly, the definition, classification and working mechanism of piezoelectric materials are briefly introduced. Then, the application of piezoelectric materials in protecting oral health and treating different oral diseases is mainly summarized and discussed (Figure 1). Meanwhile, a detailed discussion is also conducted on the treatment mechanism by which piezoelectric materials play a role, and the shortcomings are analyzed. Finally, the major challenges and future prospects facing the current applications of piezoelectric materials in the field of oral are discussed and proposed, aiming to promote the development of this emerging material in oral and provide insights and inspiration for its future clinical applications.

## 2 Piezoelectric materials

Piezoelectric materials are a type of functional material that can convert mechanical stress into electrical signals, as well as electrical signals into mechanical stress (Xu et al., 2021; Wang X. et al., 2023). The former is known as the piezoelectric effect and was first discovered by the Curie brothers in 1880 (Kapat et al., 2020; Chen S. et al., 2024). Without mechanical strain, the surface of the piezoelectric material does not show polarization, with the centers of positive and negative charges coinciding. When subjected to external force along a certain direction, causing deformation by compression or stretching, the internal polarization will occur, generating an electric current. Meanwhile, positive and negative charges will migrate and appear on the relative surfaces of the material, thus generating a piezoelectric potential and realizing the transformation of mechanical energy into electrical energy. The latter is also called inverse piezoelectric effect (Kapat et al., 2020). When the electric field is applied to the polarization direction of the piezoelectric material, the material will undergo mechanical deformation. When the electric field is removed, the deformation of the material will disappear, so as to realize the transformation from electrical energy to mechanical energy. These characteristics and good biocompatibility of piezoelectric materials lead to their wide application in biomedicine.

### 2.1 Classification

Piezoelectric materials can be roughly divided into the following four categories according to their chemical composition and structure:

(1) Inorganic piezoelectric materials. Inorganic piezoelectric materials mainly include piezoelectric crystals and piezoelectric ceramics (Xu et al., 2023). Single crystals with piezoelectric properties are called piezoelectric crystals. These crystals have a central asymmetric structure, so they have piezoelectric properties, such as quartz crystals. Due to the poor piezoelectric properties and the difficulty of preparation, the research of piezoelectric crystal in biomedical field is relatively less.

Ceramics with piezoelectric properties are called piezoelectric ceramics (Wei et al., 2024b). Compared with piezoelectric single crystal, piezoelectric ceramics have better piezoelectric properties, and have the characteristics of simple preparation and easy modification. In particular, their piezoelectric properties can be improved by morphology control, structure control, doping, defect engineering and other means, which leads to their very good application potential in the biomedical field (Wu et al., 2023). Among them, barium titanate (BaTiO_3_), zinc oxide and other piezoelectric ceramics are widely used in a variety of biomedical treatment fields (Wu et al., 2023). However, inorganic piezoelectric materials also have obvious disadvantages, such as relatively poor biocompatibility, difficult degradation and poor plasticity.

(2) Organic piezoelectric materials. Organic piezoelectric materials, also known as piezoelectric polymers, are a class of piezoelectric materials composed of organic polymers (Smith and Kar-Narayan, 2022). Polyvinylidene fluoride is the most widely used piezoelectric polymer (Mokhtari et al., 2021). In addition, polylactic acid (PLA) and its derivatives have good piezoelectric properties and have been certified and approved by the U.S. Food and Drug Administration (FDA), which may make it the first piezoelectric material to be used in clinical treatment (Zhang S. et al., 2024). Compared with inorganic piezoelectric materials, organic piezoelectric materials have better biocompatibility, flexibility and plasticity, which is convenient for subsequent processing and application, but it also has a certain disadvantage that the piezoelectric properties are relatively weak.
(3) Composite piezoelectric materials. Inorganic piezoelectric materials have relatively high dielectric constant and good piezoelectric properties, but they have high physical stiffness and poor plasticity. Although organic piezoelectric materials have certain flexibility, they are limited by low dielectric constant and poor piezoelectric properties. In view of this, researchers have developed composite piezoelectric materials, which are mainly composed of inorganic piezoelectric materials and organic piezoelectric materials, aiming to obtain piezoelectric materials with excellent comprehensive properties such as piezoelectric properties, mechanical properties and biocompatibility through composite methods (Dai et al., 2022; Dong et al., 2024; Kabakov et al., 2023). Composite piezoelectric materials can overcome the shortcomings of organic-inorganic piezoelectric materials, but it also increases the preparation process and the difficulty of preparation. Especially for the composite ratio, the wrong ratio may lead to worse piezoelectric properties.
(4) Natural piezoelectric materials. Natural piezoelectric materials refer to some proteins, tissues and bone components with piezoelectric properties from organisms (Kim et al., 2020; Bai et al., 2024). Compared with the above three synthetic piezoelectric materials, natural piezoelectric materials have the highest biological safety, but their piezoelectric properties are relatively weak, and the extraction and synthesis process is relatively complex, which seriously limits their application in the biomedical field.

### 2.2 Working mechanisms

The basis for the role of piezoelectric materials in the biomedical field is their piezoelectric effect. When piezoelectric materials undergo piezoelectric effect under the action of external mechanical force, the positive and negative charges inside the piezoelectric material separate and migrate to the surface of the material, resulting in the formation of induced potential. The contact of charged particles on the surface of piezoelectric materials with different substances results in two different working mechanisms.

(1) Electrical stimulation. When the charged particles on the surface of piezoelectric materials directly contact with biological tissues and cells, they often produce certain electrical stimulation to tissues and cells, which can well promote cell proliferation and tissue regeneration (Wu et al., 2023; Das et al., 2024). Compared with the traditional electrical stimulation therapy, piezoelectric materials get rid of the shackles of external power supply and electrode, which makes it have great application prospects in the biomedical field, especially in the field of tissue regeneration.
(2) Piezoelectric catalysis. When the charged particles on the surface of piezoelectric materials react with the surrounding medium, especially the redox reaction, reactive oxygen species (ROS) will be generated, which is called piezoelectric catalysis (Chen S. et al., 2023; Chen W. et al., 2024). The ROS produced by this piezoelectric catalytic process will cause certain damage to chemical substances, cells, tissues and bacteria, which makes it show very good application potential in decontamination, tumor treatment and antibacterial fields. At present, there are still controversies about the mechanism of piezoelectric catalysis, but the energy band theory and the screening charge effect have been recognized by many scholars. Wang et al. have discussed these two mechanisms of piezoelectric catalysis in detail, providing some guidance for people to understand the mechanism of piezoelectric catalysis (Wang K. et al., 2022).

**FIGURE 2:**
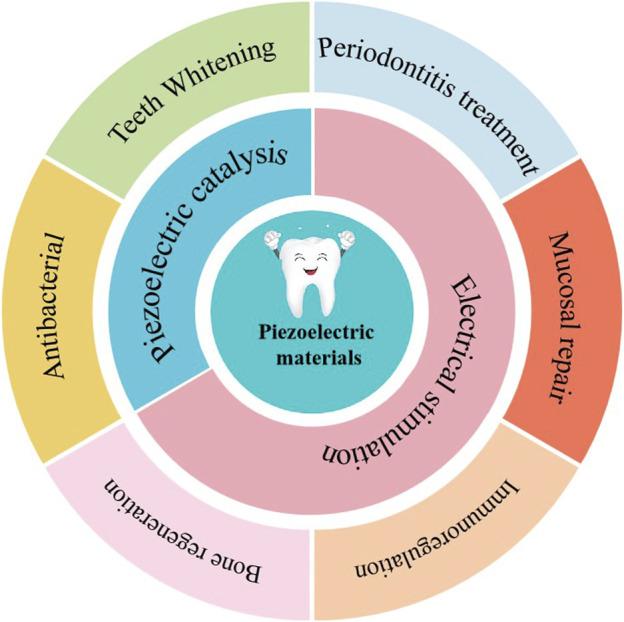
Schematic diagram of working mechanism of piezoelectric materials in maintaining oral health and treating oral diseases.

The movement of the oral cavity itself (chewing and other behaviors), daily brushing, and the direct application of ultrasound to the affected area of oral diseases are all external mechanical forces generated and received by the oral cavity in daily life. These daily external mechanical forces provide natural conditions for piezoelectric materials to excite their piezoelectric effects. When the piezoelectric effect occurs, the piezoelectric material will have the function of piezoelectric catalysis (ROS) or electrical stimulation, which makes it have great application potential in the field of maintaining oral health and treating oral diseases (Figure 2).

## 3 Teeth whitening

Brightening teeth can not only bring about the appearance of improvement, but also improve people’s self-confidence and reflect oral health problems (Goettems et al., 2021; Wang Y. et al., 2022). Common teeth whitening methods include enamel polishing, crown replacement, and tooth bleaching (Wang et al., 2020). Among them, tooth bleaching is the most widely used and effective technique, mainly achieved through the decomposition of stains on the surface of teeth by free radicals released by high concentrations of peroxides for whitening (Wang et al., 2020). However, high concentrations of hydrogen peroxide can lead to oral sensitivity, gum irritation, and enamel damage (Wang et al., 2020; Zhang et al., 2022; Kim et al., 2022). Therefore, developing a mild method of releasing ROS is a better way to achieve tooth whitening. The piezoelectric materials can generate ROS under the excitation of vibration and friction, demonstrating great potential in the field of tooth whitening. The application and mechanism of piezoelectric materials in tooth whitening is shown in Table 1.

**TABLE 1:** The application and mechanism of piezoelectric materials in tooth whitening.

Functional materials	Piezoelectric components	Function	Mechanism	Ref.
BaTiO_3_ NPs	BaTiO_3_ NPs	tooth whitening	ROS	Wang et al. (2020)
NaNbO_3_/ZnO	NaNbO_3_/ZnO	tooth cleaning and antibacterial	ROS	Sharma et al. (2022)
g-C_3_N_4-x_/Bi_2_O_3-y_	g-C_3_N_4-x_/Bi_2_O_3-y_	tooth whitening and antibacterial	ROS and surface charge	He et al. (2023)
polylactide (PLA) particles	PLA particles	tooth whitening	ROS	Deng et al. (2023)
toothbrush	PTFE electret	tooth cleaning and antibacterial	ROS	Ma et al. (2024)

**FIGURE 3:**
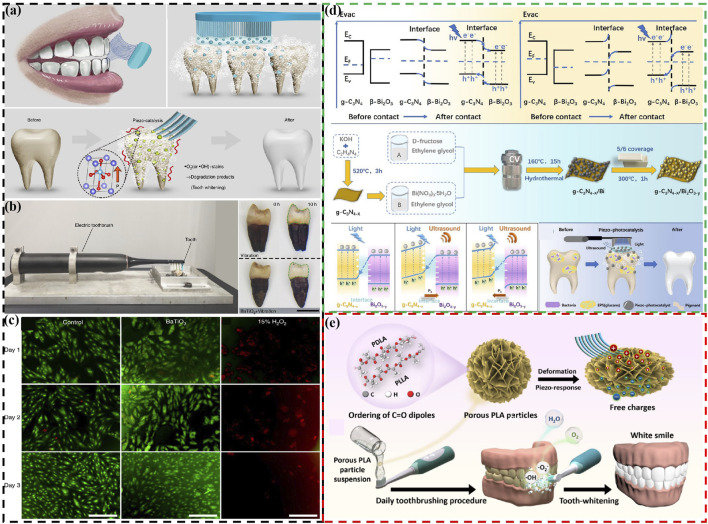
**(A)** Schematic diagram of using piezoelectric materials instead of toothpaste abrasives to achieve tooth whitening **(B)** The whitening effect of piezoelectric materials on teeth. **(C)** Cell morphology of different samples cultured for different days (Wang et al., 2020). Copyright 2020, Springer Nature. **(D)** Schematic energy band diagrams, fabrication procedure, the direct Z-scheme charge transfer mechanism of Z-scheme g-C_3_N_4-x_/Bi_2_O_3-y_ heterostructures, and simplified schematic for piezo-photocatalytic treatment of tooth whitening and biofilm eradication using Z-scheme g-C_3_N_4-x_/Bi_2_O_3-y_ heterostructures (He et al., 2023). Copyright 2023, Royal Society of Chemistry. **(E)** Schematic diagram of biodegradable polylactic acid particles used for piezoelectric catalytic teeth whitening (Deng et al., 2023). Copyright 2023, American Chemical Society.

Wang et al. (2020) report a non-destructive, harmless, and convenient tooth whitening strategy based on piezoelectric catalytic effect . Using polarized piezoelectric BaTiO_3_ to replace abrasives in toothpaste, combine with electric toothbrush vibration, can effectively remove staining compounds and achieve teeth whitening (Figures 3A, B). Meanwhile, they also found that BaTiO_3_ has lower cytotoxicity compared to commonly use hydrogen peroxide whitening agents (Figure 3C) and does not damage the surface of teeth. However, in order to fully utilize the piezoelectric catalytic whitening ability of BaTiO_3_, specialized equipment is needed to add the electric polarization step, which significantly affects its use. Meanwhile, the piezoelectric catalytic ability of a single BaTiO_3_ is also very weak. To further enhance the piezoelectric catalytic capability for better teeth whitening effects, different approaches have been proposed. Sharma et al. develop a NaNbO_3_/ZnO heterojunction piezoelectric catalyst for teeth whitening, showing reaction rates 8.5 times higher than ZnO and 1.7 times higher than NaNbO_3_ (Sharma et al., 2022). He et al. combine photocatalysis and piezoelectric catalysis for teeth whitening by designing Z-scheme g-C_3_N_4-x_/Bi_2_O_3-y_ heterostructures (Figure 3D) (He et al., 2023). This design enhances internal charge separation, significantly boosting its piezo-photocatalytic efficiency and achieving degradation rates approximately four times than piezoelectric catalysis alone and 2.6 times than photocatalysis alone for typical food colorants.

Although there is extensive research on the use of inorganic piezoelectric materials for teeth whitening, the safety of certain ions such as Ba, Nb, and Bi in these materials still requires further investigation. Specifically, using these inorganic piezoelectric materials directly as abrasives in toothpaste may pose biosecurity concerns as they could enter the human body through the oral cavity. Based on this, relatively safe biodegradable piezoelectric materials are also used in teeth whitening. Deng et al. develop biodegradable PLA particles for teeth whitening (Figure 3E) (Deng et al., 2023). Furthermore, researchers have also used piezoelectric materials as fillers or coatings, such as for toothbrush bristles, to achieve teeth whitening (Ma et al., 2024). This prevents piezoelectric materials from entering the human body as toothpaste abrasives.

## 4 Oral disease treatment

Under the mechanical forces exerted during daily oral activities such as chewing, speaking, and brushing teeth, piezoelectric materials can undergo mechanical deformation effectively, thereby activating their piezoelectric effect to achieve the goal of treating oral diseases. Table 2 summarizes the application and mechanism of piezoelectric materials in maintaining oral health and treating oral diseases.

**TABLE 2:** The treatment and mechanism of piezoelectric materials in different oral diseases.

Functional materials	Piezoelectric components	Function	Mechanism	Ref.
NaNbO_3_/ZnO	NaNbO_3_/ZnO	tooth cleaning and antibacterial	ROS	Sharma et al. (2022)
g-C_3_N_4-x_/Bi_2_O_3-y_	g-C_3_N_4-x_/Bi_2_O_3-y_	tooth whitening, antibacterial	ROS and surface charge	He et al. (2023)
toothbrush	PTFE electret	tooth cleaning and antibacterial	ROS	Ma et al. (2024)
BaTiO_3_/chitosan multiporous piezoelectric coating	BaTiO_3_ NPs	antibacterial and mineralization effects	ROS and surface charge	Wei et al. (2024c)
Al-SrTiO_3_/TiO_2_ nanotubes piezoelectric coating	Al-SrTiO_3_/TiO_2_ nanotubes	antibacterial and promote osteogenic activity	ROS and Sr^2+^	Pan et al. (2024)
piezoelectric PMMA dentures	BaTiO_3_ NPs	antifungal activity	ROS	Montoya et al. (2021b)
dental piezoelectric resin composites	BaTiO_3_ NPs	antibacterial and mineralization effects	ROS and surface charge	Montoya et al. (2021a)
orthodontic invisible appliance	BaTiO_3_ NPs	antibacterial effect	ROS	Shi et al. (2023)
BaTiO_3_/P(VDF-TrFE) electroactive film	BaTiO_3_/P(VDF-TrFE)	treatment of periodontitis	ROS and electrical stimulation	Song et al. (2024)
Injectable piezoelectric hydrogel	BaTiO_3_ NPs	treatment of periodontitis	ROS and electrical stimulation	Roldan et al. (2023)
Piezoelectric hydrogel	Cubic BaTiO_3_ NPs	injured tissue regeneration in periodontitis	Electrical stimulation	Liu X. et al. (2024)
P(VDF-TrFE) piezoelectric film (2 wt% SrCl_2_)	P(VDF-TrFE)	repair and regeneration of dentin tissue	Electrical stimulation and Sr^2+^	Li J. et al. (2024)
VDF-TeFE piezoelectric film	VDF-TeFE	re-generation of oral mucosa	Electrical stimulation	Chernova et al. (2024)
VDF-TeFE piezoelectric film (Cu^2+^)	VDF-TeFE	re-generation of oral mucosa and antibacterial effect	Electrical stimulation and Cu^2+^	Badaraev et al. (2020)

### 4.1 Antibacterial

The oral microbiota includes a variety of bacteria, fungi, and viruses, which form a complex ecosystem in the oral environment and interact with the host to positively or negatively impact oral health (Lu et al., 2019). When oral microorganisms proliferate excessively, they can form dental plaque and calculus, leading to the occurrence and development of oral diseases such as gingivitis and periodontitis (Sedghi et al., 2021). Especially, some studies have shown that oral microbiota is also associated with other systemic diseases (Peng et al., 2022). Therefore, effective oral sterilization can not only remove dental plaque and tartar, reduce the number and types of bacteria, but also reduce the risk of oral inflammation and maintain the health of teeth and gums.

**FIGURE 4:**
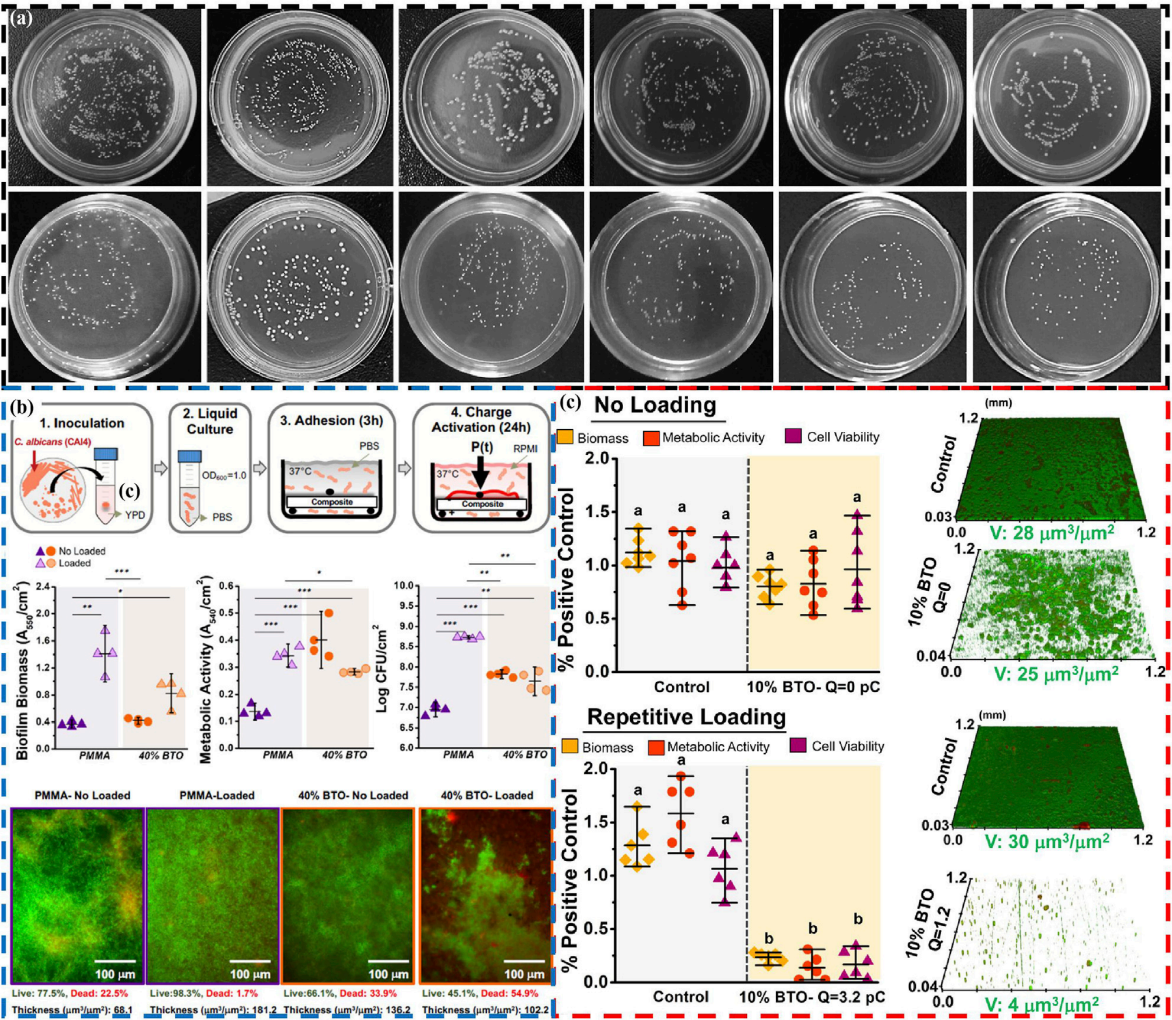
**(A)** Antibacterial activity of Z-scheme g-C_3_N_4-x_/Bi_2_O_3-y_ heterostructures: Photos of planktonic bacterial colonies and bacterial colonies from disrupted biofilms under different conditions and materials (He et al., 2023). Copyright 2023, Royal Society of Chemistry. **(B)** The antifungal effect of piezoelectric PMMA dentures: Schematic of the biofilm model, microbiological evaluations (biofilm biomass, metabolic activity and cell viability), and fluorescence microscopy z-stack images of *C. albicans* biofilms (Montoya et al., 2021b). Copyright 2021, American Chemical Society. **(C)** Antibacterial activity of dental piezoelectric resin composites: Biofilm biomass, metabolic activity, cell viability, and CLSM images of *S. mutans* biofilms under no loading and repetitive loading (Montoya et al., 2021a). Copyright 2021, American Chemical Society.

Piezoelectric materials are used in the field of oral antimicrobials in two main ways: one approach involves direct use of piezoelectric materials, similar to toothpaste grinding agent, for oral antimicrobial (Sharma et al., 2022; He et al., 2023). Sharma et al. also directly apply NaNbO_3_/ZnO piezoelectric material used for tooth whitening to antibacterial applications (Sharma et al., 2022), but only briefly studied its antibacterial ability against *Escherichia coli*, lacking in-depth research, especially its antibacterial performance against major pathogenic bacteria in the oral cavity. In response to this, He et al. apply Z-scheme g-C_3_N_4-x_/Bi_2_O_3-y_ heterostructures with piezoelectric photocatalytic ability to oral antibacterial applications (He et al., 2023). It could not only directly kill planktonic *S. mutans* (*Streptococcus mutans*) (Figure 4A) (Antibacterial rate: 63%), but also kill *S. mutans* embedded in the biofilm formed by *S. mutans* (Antibacterial rate: 48.2%). In addition, the antibacterial mechanism of the g-C_3_N_4-x_/Bi_2_O_3-y_ is also proposed, mainly by stimulating the production of ROS in the environment, leading to bacterial death. Meanwhile, due to the presence of positive charges on the surface of g-C_3_N_4-x_/Bi_2_O_3-y_, they can better adsorb negatively charged *S. mutans*, thereby enhancing their piezoelectric photocatalytic ability and resulting in high antibacterial performance.

Another approach targets the susceptibility of dental implants to bacterial infection-induced damage (Zhou et al., 2024; Jayasree et al., 2024), using piezoelectric materials as fillers or coatings on dental implants to achieve antimicrobial effects. Wei et al. provide a new strategy to enhance the antibacterial effect (90.41%) of implants using biocompatible BaTiO_3_/chitosan multiporous surface piezoelectric coating (Wei et al., 2024c). The main reasons for this high antibacterial effect are as follows: On the one hand, the generation of ROS under ultrasonic stimulation in piezoelectric coating kills bacteria. On the other hand, the contact potential difference of bacteria by surface charges on piezoelectric coating leads to the production of ROS inside the bacteria, which also kills bacteria. Meanwhile, the coating promotes increased deposition of hydroxyapatite and adhesion of plasma albumin, facilitated by its abundant positive charges and numerous pores on the surface. In addition, Pan et al. design a surface ultrasonic response coating of Al^3+^ ion doped strontium titanate/titanium dioxide nanotubes (Al-SrTiO_3_/TiO_2_ nanotubes) for antibacterial properties of dental implants (Pan et al., 2024). The introduction of Al^3+^ ions induces oxygen vacancies, destroys the lattice of SrTiO_3_, and enables Al-SrTiO_3_/TiO_2_ nanotubes to produce more ROS, thereby achieving efficient antibacterial (*P. gingivalis*: 80.4% and *F. nucleatum*: 82.1%) and inhibiting the growth of biofilms. In particular, the presence of Sr^2+^ ions in SrTiO_3_ can effectively promote osteogenic activity and facilitate the formation of rigid bone fusion between the implant surface and alveolar bone. The multifunctionality of piezoelectric materials increase through ion doping or the addition of functional ions has important guiding significance for the application of piezoelectric materials in the oral field.

In addition to the aforementioned primary applications, piezoelectric materials are also used as fillers for antibacterial purposes in dentures (Montoya et al., 2021b), dental composite restorative materials (Montoya et al., 2021a), and orthodontic appliances (Shi et al., 2023). Montoya et al. demonstrate for the first time that piezoelectric nanoparticles of BaTiO_3_ can serve as fillers for dentures, achieving antifungal effects (Biofilm volume decreased by 43%) (Figure 4B) (Montoya et al., 2021b). The main reason for achieving antifungal effects may be the interaction (surface charge to induce the production of ROS) between the surface charges of BaTiO_3_ and fungi, leading to fungal death. The research team also apply BaTiO_3_ nanoparticles as fillers to dental composite repair materials (Montoya et al., 2021a). The BaTiO_3_ can not only impart antibacterial (up to 90%) to composite materials (Figure 4C), but also promote the remineralization of dental tissues. Under the stimulation of mechanical external forces, a piezoelectric charge is generated on the surface of the composite material, which interacts with bacteria through electrostatic interactions, leading to bacterial repulsion, preventing further adhesion, and ultimately inhibiting the growth of biofilms. Meanwhile, these charges also promote the production of ROS in cells, thereby killing bacteria. And its mineralization mechanism is also due to the presence of surface charges of piezoelectric materials promoting the nucleation of calcium phosphate, leading to its re-mineralization. Here, piezoelectric materials may also have other functions such as promoting tooth tissue remineralization during antibacterial processes, which also needs further development and utilization.

### 4.2 Treatment of periodontitis

Periodontitis (PD) is a local chronic inflammatory disease of periodontal tissue caused by pathogenic microorganisms (Mi et al., 2024). Under the continuous stimulation of bacteria and their metabolites, periodontal tissue undergoes inflammatory reactions and immune regulation imbalances, ultimately leading to structural damage of periodontal tissue such as alveolar bone loss (Huang et al., 2020). In the pathogenesis of PD, in addition to pathogenic bacteria, host immune response is also a key mediator of periodontal damage (Lai et al., 2023; Yang et al., 2021).

**FIGURE 5:**
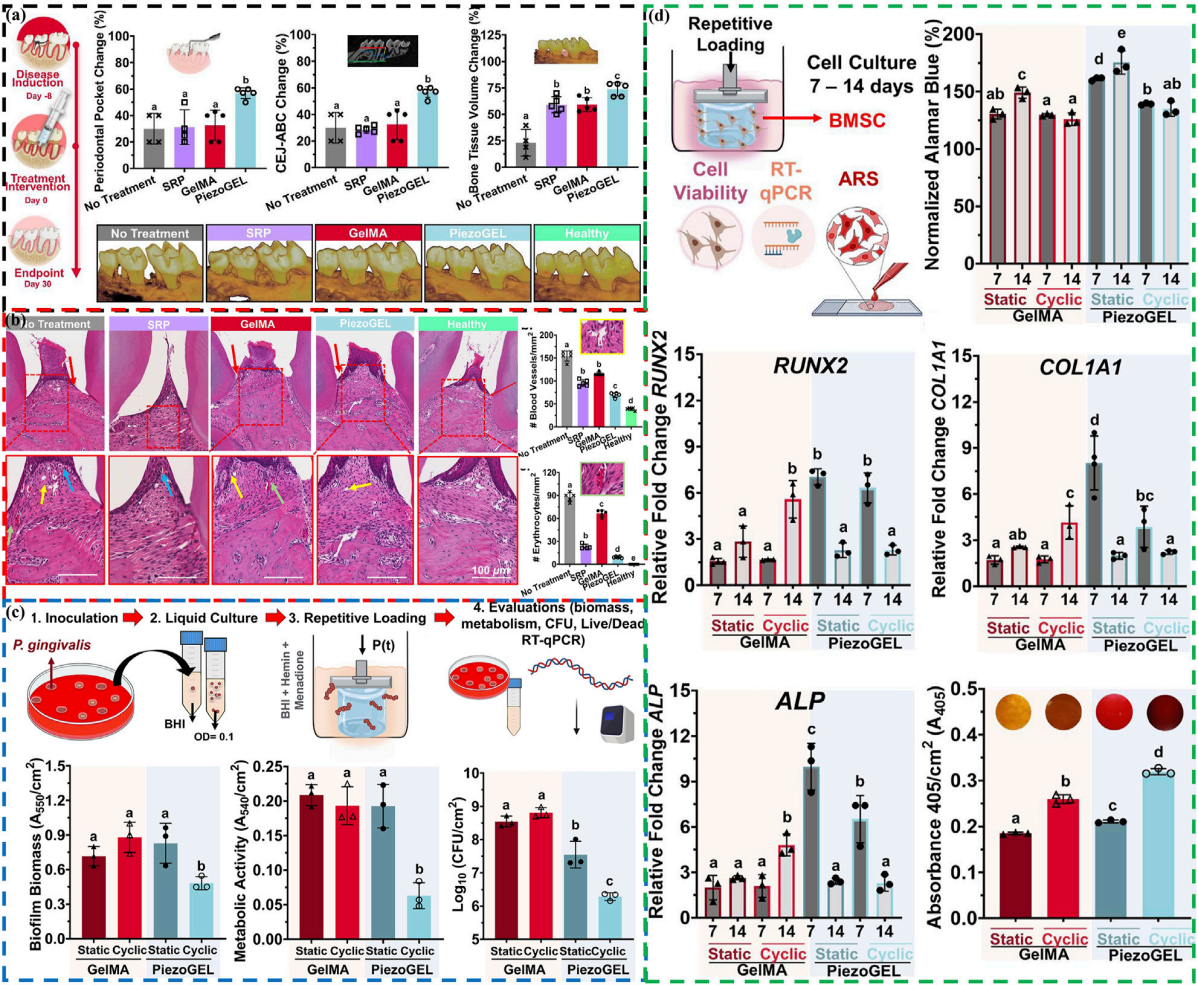
**(A)** Periodontal bone tissue regeneration evaluation: Schematic representation of the ligature-induced model, changes of periodontal pocket depth, cementum-enamel junction to alveolar bone crest distance and bone volume, and reconstructed 3D micro-CT images of the maxillary alveolar bone. **(B)** Histological evaluation: H&E images of periodontal tissue sections, number of blood vessels and erythrocytes quantified in the regenerated area. **(C)** Antibacterial evaluation of PiezoGEL hydrogels *in vitro*: Schematics of the model, biofilm biomass, metabolic activity, and cell viability. **(D)** Osteogenic differentiation evaluation of PiezoGEL hydrogels *in vitro*: Schematics of the model, changes of Cell viability (RUNX2, COL1A1, ALP and ECM minerals) (Roldan et al., 2023). Copyright 2023, American Chemical Society.

Current treatment strategies mainly focus on reducing bacterial adhesion or killing bacteria (Mei et al., 2024), as well as regulating the host immunity to inhibit the progression of chronic inflammation (Zheng et al., 2023; Peng et al., 2024). For alveolar bone resorption, a prominent feature of periodontal disease, the treatment plan is to promote bone regeneration to achieve the repair of missing bone (Luan et al., 2023; Song et al., 2024; Dong et al., 2023). Based on this, Roldan et al. develop an injectable piezoelectric hydrogel (PiezoGEL), comprising methacryloyl gelatin (GelMA) and biocompatible BaTiO_3_ piezoelectric filler (BTO), which generates charges in response to biomechanical vibrations like chewing and movement (Roldan et al., 2023). This new type of PiezoGEL exhibits good biological activity, which can promote bone tissue regeneration (Figures 5A, B) and achieve high antibacterial effects (Figure 5C). PiezoGEL significantly reduce pathogenic biofilm biomass (∼41%), metabolic activity (∼75%), and viable cell count (∼2-3 log) compared to BTO-free hydrogels *in vitro*. The antibacterial mechanism is mainly due to the surface charge of BTO and the generation of ROS. Molecular analysis of the antibacterial effect attributes it to decreased cell adhesion (downregulation of *porP* and *fimA*) and increased oxidative stress (upregulation of *oxyR*). The primary mechanism of bone tissue regeneration is attributed to electrical stimulation generated by the surface charge of BTO, which promotes differentiation and proliferation of bone marrow stem cells (BMSCs) and facilitates the formation of new bone tissue (upregulating *RUNX2*, *COL1A1*, and *ALP*) (Figure 5D). Additionally, these charges also stimulate necessary vascularization for bone regeneration. Especially, *in vivo* experiments have shown that PiezoGEL can effectively reduce periodontal inflammation and increase bone tissue regeneration, thus having good application prospects in the treatment of periodontal disease.

**FIGURE 6:**
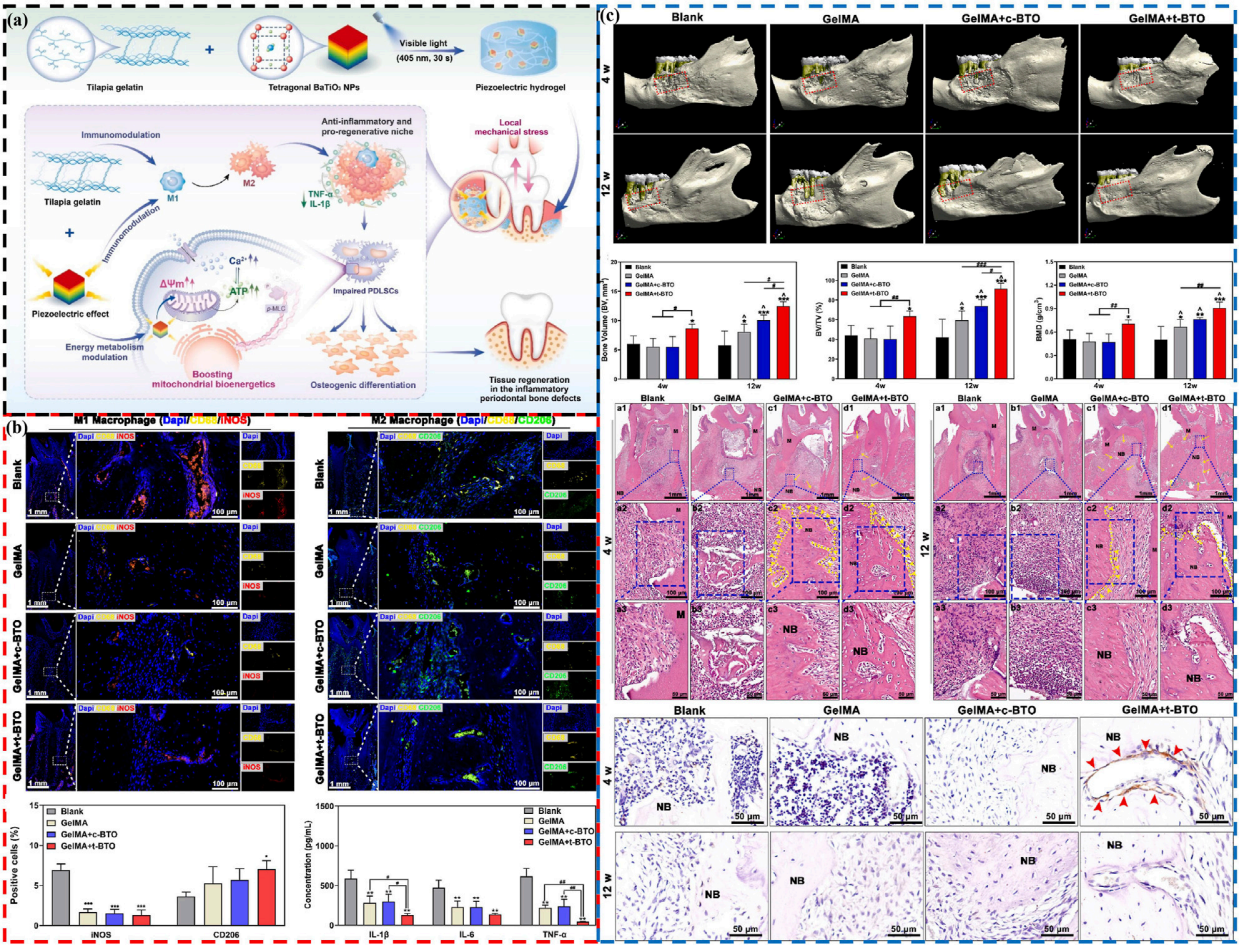
The piezoelectric hydrogel’s role in promoting osteogenesis and immunomodulation in PD by activating bioenergetics. **(A)** Schematic diagram of piezoelectric hydrogel preparation and treatment of PD. **(B)** Piezoelectric stimulation modulates M1/M2 polarization of macrophages: Representative fluorescence images and positive percentage of M1 and M2 macrophages, and salivary inflammatory factors after 12 weeks **(C)** The piezoelectric hydrogel enhances bone regeneration in periodontal defects associated with PD: Micro-CT 3D construction images, new bone volume, bone volume/total volume fraction, bone mineral density, H&E staining images and p-MLC immunohistochemistry images at 4 weeks and 12 weeks (Liu X. et al., 2024). Copyright 2024, Elsevier.

The impaired differentiation ability of resident cells and the disruption of immune microenvironment in PD seriously affect the regeneration of alveolar bone (Li et al., 2022; Li et al., 2023). Research has found that electrical stimulation can reduce inflammation, regulate macrophage polarization and promote bone regeneration by improving mitochondrial function and inducing more adenosine triphosphate (ATP) synthesis (Luo et al., 2024; Hollenberg et al., 2021). Inspired by this, Liu et al. develop a wireless piezoelectric stimulation system using a piezoelectric hydrogel composed of tetragonal BaTiO_3_ nanoparticles (t-BTO NPs) and tilapia fish gelatin hydrogel to activate bioenergetics for injured tissue regeneration in PD (Figure 6A) (Liu X. et al., 2024). Under mechanical activation, the triggered piezoelectric potential induces osteogenic differentiation of inflammatory periodontal ligament stem cells (PDLSCs) by regulating energy metabolism and enhancing ATP synthesis. Additionally, under the synergistic action of piezoelectric stimulation and the intrinsic anti-inflammatory activity of the hydrogel, macrophage polarization is shifted from the pro-inflammatory M1 phenotype to the anti-inflammatory M2 phenotype, promoting osteogenesis (Figure 6B). This ultimately achieve *in situ* tissue regeneration of rat periodontitis bone defects (Figure 6C), paving new pathways for treating PD and other immune-related bone defects through piezoelectric stimulation to regulate energy metabolism and immune modulation.

In addition, for the repair and regeneration of dentin tissue, Li et al. design a P(VDF-TrFE) piezoelectric film with 2 wt% SrCl_2_ addition (Li J. et al., 2024). Piezoelectric film creates an electric microenvironment conducive to recruiting dental pulp stem cells (DPSCs) and guiding their differentiation into odontoblasts during everyday activities like chewing and speaking. Moreover, the gradual release of Sr^2+^ ions from the film promotes the odonto-differentiation of DPSCs. The mutual assistance of electrical stimulation and Sr^2+^ ions enables the regeneration of dentin tissue, providing a new treatment approach for the repair of damaged teeth such as alveolar bone loss.

### 4.3 Oral mucosal regeneration

Oral mucosa not only serves as a protective barrier inside the mouth, effectively preventing bacteria and viruses from invading deep tissues, but also maintains comfort and normal function in the mouth through sensory functions (Moutsopoulos and Konkel, 2018; Zhao et al., 2022). Therefore, wound healing of the oral mucosa is an important measure to prevent oral diseases and promote oral health.

**FIGURE 7:**
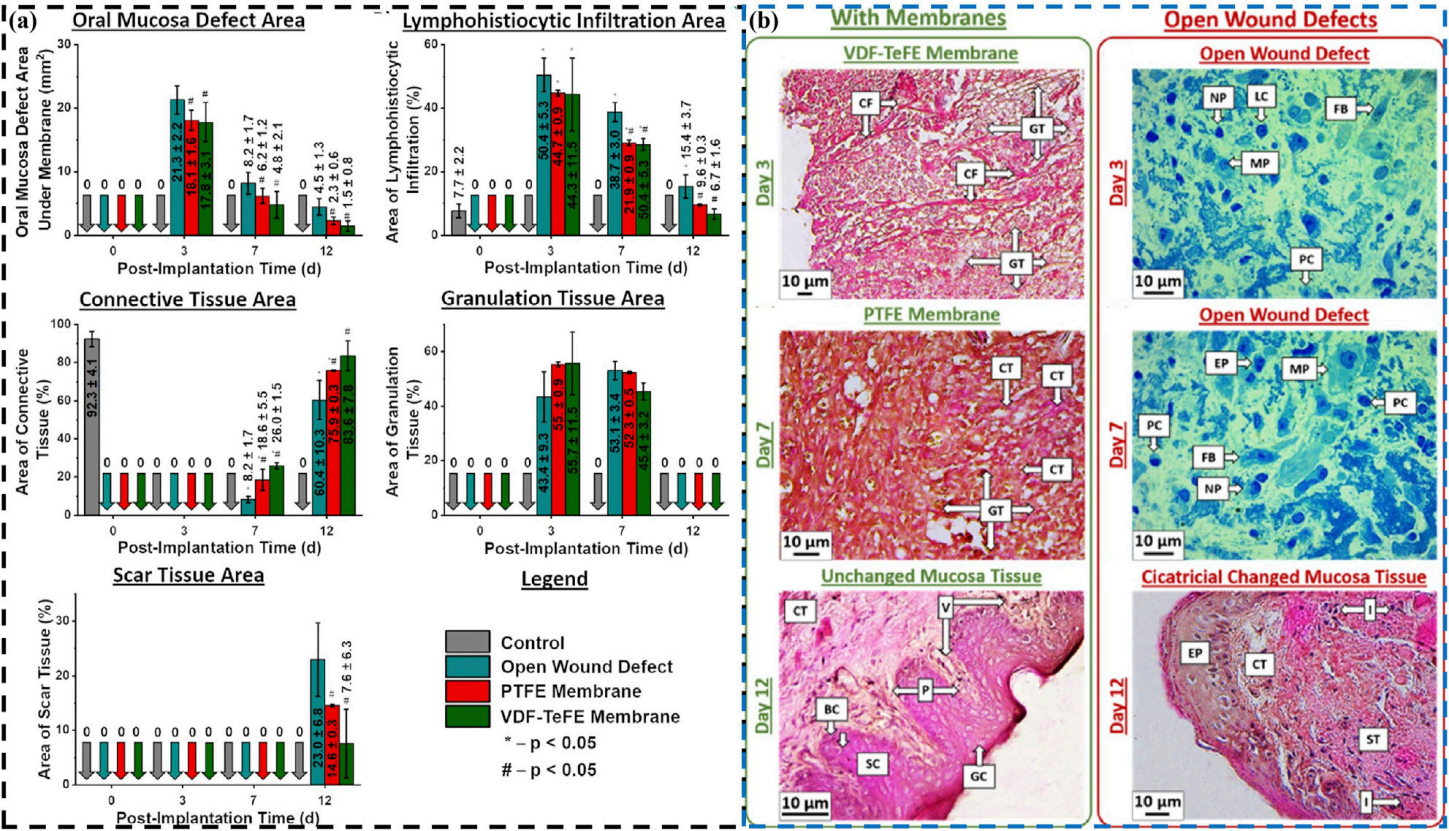
**(A)** The wound defect healing of PTFE (red) and VDF-TeFE (green): Area of the oral mucosa defect under the membranes, and specific area of lymphohistiocytic infiltration, connective tissue, granulation tissue and scar tissue. **(B)** Histological examination of the oral mucosa: Changes in wound defect areas (connective tissue fibers, cellular infiltration, granulation tissue, mucosa and cicatricial) under different films at different times (day 3, day 7 and day 12) (Chernova et al., 2024). Copyright 2024, American Chemical Society.

Piezoelectric materials can promote cell growth and repair due to their unique electrical stimulation effects, especially during wound healing, where appropriate electrical stimulation can accelerate tissue repair and regeneration (Dai et al., 2024; Liu Q. et al., 2024; Ren et al., 2024). Chernova et al. conduct a comparative study on two different types of polymeric membranes in promoting wound healing processes in oral mucosa: dielectric poly(tetrafluoroethylene) (PTFE) membrane and piezoelectric membrane of vinylidene fluoride and tetrafluoroethylene (VDF-TeFE) (Chernova et al., 2024). Comparative experiments have shown that piezoelectric VDF-TeFE membranes have high oral mucosal regeneration ability (Figure 7). Badaraev et al. also confirm that piezoelectric polymer membranes (copolymer of vinylidene fluoride with tetrafluoroethylene) can be used for oral mucosal regeneration (Badaraev et al., 2020), especially by coating them with antibacterial Cu^2+^ ion coatings, which can further enhance their oral mucosal regeneration ability. These indicate that piezoelectric materials have broad application prospects in oral mucosal repair.

## 5 Treatment mechanism

According to the role of the above piezoelectric materials in the treatment of oral diseases and the working mechanism of piezoelectric materials, the treatment mechanism (Table 2) is as follows:

(1) The mechanism of piezoelectric materials in tooth whitening is mainly to generate charges on the surface under mechanical force stimulation, which reacts with the external environment, generates ROS (Piezoelectric catalysis), and realizes the degradation of tooth surface staining and stains, achieving the whitening effect (Wang et al., 2020; Deng et al., 2023).
(2) The inhibitory mechanism of piezoelectric materials on oral microbiota is similar to that of teeth whitening, mainly relying on the reaction (Piezoelectric catalysis) of piezoelectric materials with surrounding media under external mechanical forces such as ultrasound to generate ROS (He et al., 2023; Wei et al., 2024c; Pan et al., 2024). The ROS can cause bacterial death through pathways such as oxidative stress, lipid peroxidation, protein dysfunction, and DNA damage. However, another antibacterial property of piezoelectric materials has also been proposed, which is the charge generated on the surface of the piezoelectric material, which generates a certain stimulation on cells, leading to the production of ROS inside bacteria and ultimately causing bacterial death (Montoya et al., 2021a; Wei et al., 2024c). In addition, the surface charge of piezoelectric materials may also interact with the surface charge of bacteria, leading to bacterial death or inhibiting the formation of bacterial biofilms (Montoya et al., 2021a). Therefore, further research is needed on the antibacterial mechanism of piezoelectric materials to find their biological mechanisms.
(3) In the treatment of PD, one mechanism is that piezoelectric materials can inhibit bacteria to treat PD, and its treatment mechanism is antibacterial mechanism (Roldan et al., 2023). Another mechanism is to generate electric charges on the surface of piezoelectric materials, which can produce electrical stimulation (Roldan et al., 2023; Liu X. et al., 2024). Under the action of electrical stimulation, it can improve mitochondrial function and induce more ATP synthesis to alleviate inflammatory response, promote osteoblast differentiation, regulate macrophage polarization to control the immune microenvironment, and ultimately achieve regeneration and repair of damaged dental bone tissues.
(4) The mechanism of oral mucosal repair is basically consistent with the mechanism of treating PD, mainly through the electrical stimulation effect of piezoelectric materials (Chernova et al., 2024; Badaraev et al., 2020). Under the action of electrical stimulation, it improves the function of intracellular mitochondria, increases energy production of cells (such as ATP synthesis), and promotes the improvement of cell metabolism and repair ability. In addition, reducing local inflammatory response and controlling the release of inflammatory mediators under electrical stimulation can help reduce pain and discomfort during wound healing process.

Based on the above analysis, the fundamental role of piezoelectric materials in treating oral diseases lies in their ability to generate electric charges and fields when subjected to mechanical force or pressure. It is precisely this electrical stimulation that facilitates the treatment of oral diseases. However, further in-depth research is needed on the detailed effects and mechanisms of electrical stimulation on the internal cells or tissues of living organisms, which has certain guiding significance for the application of piezoelectric materials. In addition, it can also be found that antibacterial is the basis and auxiliary for the treatment of many oral diseases, and the addition of antibacterial process can increase the therapeutic effect on other oral diseases.

## 6 Shortcomings and improvements

Piezoelectric materials have shown great potential in the treatment of oral diseases, but there are still shortcomings that need improvement. The specific details are as follows:

(1) The bottleneck limiting the application of piezoelectric materials is that their piezoelectric performance is too weak. Chemical preparation methods such as ion doping (Pan et al., 2024; Wang X. et al., 2022; Chen T. et al., 2023) and heterojunction (Sharma et al., 2022; He et al., 2023) can improve piezoelectric performance or develop new piezoelectric materials with high voltage capability (Zhang H. et al., 2024).
(2) At present, the application of piezoelectric materials in oral treatment is too limited, which is a typical acoustic dynamic therapy (Roldan et al., 2023; Chernova et al., 2024). The single treatment method may affect its effectiveness, and it can be combined with other treatment methods such as photothermal, photoelectric, and drug methods to achieve multifunctional treatment of oral diseases and improve efficacy.
(3) The mechanism of piezoelectric materials in tooth whitening has been basically understood (Wang et al., 2020), but the mechanism of their effects in the treatment of other oral diseases is not clear and and controversial (Montoya et al., 2021a; Wei et al., 2024c; Montoya et al., 2021b). The latest biological methods such as proteomics and metabolomics can be used to further study and explore their deep therapeutic mechanisms.
(4) Piezoelectric materials have been widely studied in tumor treatment and have shown good therapeutic effects (Zheng et al., 2024; Zhang L. et al., 2024; Hao et al., 2024). However, there is currently no application of piezoelectric materials in the treatment of oral cancer, which is a new direction for the future application of piezoelectric materials in the field of oral treatment.
(5) The biological safety and metabolic process of piezoelectric materials entering the body are the real problems that need to be solved, and are the prerequisites for their clinical application.In particular, the metal ions contained in some inorganic piezoelectric materials may have specific toxicity to the human body (Bhasin et al., 2019). Therefore, exploring the toxicological research of piezoelectric materials is of utmost importance.

## 7 Conclusion and perspective

This review comprehensively summarizes and classifies the research on piezoelectric materials in protecting oral health and treating oral diseases in the past 5 years. Among them, representative studies are discussed in detail and compared, and their underlying therapeutic mechanisms and innovation points are explored. Then, each mechanism of the application of piezoelectric materials in the field of oral cavity is discussed and analyzed in detail, which provides a new treatment idea and a new method for the treatment of oral diseases by piezoelectric materials in the future. Finally, the current deficiencies of piezoelectric materials in the field of oral therapy are analyzed, and improvement measures are proposed to provide solid support for the real clinical application of piezoelectric materials.

Although the application of piezoelectric materials in this field is still in the research and development stage, their high-performance characteristics and multi-functional application potential provide a new prospect for personalized treatment and precision medicine for oral health in the future. With the continuous advancement of technology and in-depth exploration of theories, it is believed that the application of piezoelectric materials in the field of oral disease treatment will become more and more extensive, bringing people a healthier and more comfortable oral care experience.

## References

[B1] Badaraev A. D. Koniaeva A. Krikova S. A. Shesterikov E. V. Bolbasov E. N. Nemoykina A. L. (2020). Piezoelectric polymer membranes with thin antibacterial coating for the regeneration of oral mucosa. Appl. Surf. Sci. 504, 144068. 10.1016/j.apsusc.2019.144068

[B2] Bai Y. Meng H. Li Z. Wang Z. L. (2024). Degradable piezoelectric biomaterials for medical applications. MedMat 1 (1), 40–49. 10.1097/mm9.0000000000000002

[B3] Baker J. L. Welch J. L. M. Kauffman K. M. McLean J. S. He X. (2024). The oral microbiome: diversity, biogeography and human health. Nat. Rev. Microbiol. 22, 89–104. 10.1038/s41579-023-00963-6 37700024 PMC11084736

[B4] Bhasin A. K. K. Chauhan P. Chaudhary S. (2019). A novel sulfur-incorporated naphthoquinone as a selective “turn-on” fluorescence chemical sensor for rapid detection of Ba^2+^ ion in semi-aqueous medium. Sens. Actuator. B Chem. 294, 116–122. 10.1016/j.snb.2019.04.098

[B5] Botelho J. Mascarenhas P. Viana J. Proença L. Orlandi M. Leira Y. (2022). An umbrella review of the evidence linking oral health and systemic noncommunicable diseases. Nat. Commun. 13, 7614. 10.1038/s41467-022-35337-8 36494387 PMC9734115

[B6] Chen S. Tong X. Huo Y. Liu S. Yin Y. Tan M. (2024a). Piezoelectric biomaterials inspired by nature for applications in biomedicine and nanotechnology. Adv. Mater. 36, 2406192. 10.1002/adma.202406192 39003609

[B7] Chen S. Zhu P. Mao L. Wu W. Lin H. Xu D. (2023a). Piezocatalytic medicine: an emerging frontier using piezoelectric materials for biomedical applications. Adv. Mater. 35, 2208256. 10.1002/adma.202208256 36634150

[B8] Chen T. Zhu Z. Chen F. Hu C. Huang H. (2023b). Low-valence B-site cation substitution triggering polarization and oxygen vacancy enhancement for elevating piezocatalytic activity on Bi_2_MoO_6_ . Appl. Surf. Sci. 616, 156467. 10.1016/j.apsusc.2023.156467

[B9] Chen W. Chen Q. Song F. Hua M. Chang M. Feng W. (2024b). Engineering heterostructured piezoelectric nanorods with rich oxygen vacancy-mediated piezoelectricity for ultrasound-triggered piezocatalytic cancer therapy. Adv. Funct. Mater., 2405929. 10.1002/adfm.202405929

[B10] Chernova U. V. Varakuta E.Yu. Koniaeva A. D. Leyman A. E. Sagdullaeva S. A. Plotnikov E. (2024). Piezoelectric and dielectric electrospun fluoropolymer membranes for oral mucosa regeneration: a comparative study. ACS Appl. Mater. Interfaces 16, 20245–20259. 10.1021/acsami.4c01867 38607352

[B11] Dai J. Shao J. Zhang Y. Hang R. Yao X. Bai L. (2024). Piezoelectric dressings for advanced wound healing. J. Mater. Chem. B 12, 1973–1990. 10.1039/d3tb02492j 38305583

[B12] Dai X. Yao X. Zhang W. Cui H. Ren Y. Deng J. (2022). The osteogenic role of barium titanate/polylactic acid piezoelectric composite membranes as guiding membranes for bone tissue regeneration. Int. J. Nanomed. 17, 4339–4353. 10.2147/ijn.s378422 PMC9491370 36160471

[B13] Das K. K. Basu B. Maiti P. Dubey A. K. (2024). Interplay of piezoelectricity and electrical stimulation in tissue engineering and regenerative medicine. Appl. Mater. Today 39, 102332. 10.1016/j.apmt.2024.102332

[B14] Deng S. Zhang Y. Qiao Z. Wang K. Ye L. Xu Y. (2023). Hierarchically designed biodegradable polylactide particles with unprecedented piezocatalytic activity and biosafety for tooth whitening. Biomacromolecules 24, 797–806. 10.1021/acs.biomac.2c01252 36642871

[B15] Ding Y. Zhu Z. Zhang X. Wang J. (2024). Novel functional dressing materials for intraoral wound care. Adv. Healthc. Mater., 2400912. 10.1002/adhm.202400912 38716872

[B16] Dong H. Zhu Z. Li Z. Li M. Chen J. (2024). Piezoelectric composites: state-of-the-art and future prospects. JOM 76, 340–352. 10.1007/s11837-023-06202-w

[B17] Dong Z. Lin Y. Xu S. Chang L. Zhao X. Mei X. (2023). NIR-triggered tea polyphenol-modified gold nanoparticles-loaded hydrogel treats periodontitis by inhibiting bacteria and inducing bone regeneration. Mater, Des. 225, 111487. 10.1016/j.matdes.2022.111487

[B18] Goettems M. L. Fernandez M. d.S. Donassollo T. A. Donassollo S. H. Demarco F. F. (2021). Impact of tooth bleaching on oral health-related quality of life in adults: a triple-blind randomised clinical trial. J. Dent. 105, 103564. 10.1016/j.jdent.2020.103564 33359042

[B19] Hao Z. Guo S. Tu W. Wang Q. Wang J. Zhang X. (2024). Piezoelectric catalysis induces tumor cell senescence to boost chemo-immunotherapy. Small 20, 2309487. 10.1002/smll.202309487 38197548

[B20] He J. Cui S. Hou Y. Liu S. Zhang Z. Zhao M. (2023). Bifunctional defect mediated direct Z-scheme g-C_3_N_4-x_/Bi_2_O_3-y_ heterostructures with enhanced piezo-photocatalytic properties for efficient tooth whitening and biofilm eradication. J. Mater. Chem. B 11, 7103–7116. 10.1039/d3tb01044a 37417809

[B21] Hollenberg A. M. Huber A. Smith C. O. Eliseev R. A. (2021). Electromagnetic stimulation increases mitochondrial function in osteogenic cells and promotes bone fracture repair. Sci. Rep. 11 (1), 19114. 10.1038/s41598-021-98625-1 34580378 PMC8476611

[B22] Huang X. Xie M. Xie Y. Mei F. Lu X. Li X. (2020). The roles of osteocytes in alveolar bone destruction in periodontitis. J. Transl. Med. 479, 18. 10.1186/s12967-020-02664-7 PMC7733264 33308247

[B23] Jayasree A. Cartmell S. Ivanovski S. Gulati K. (2024). Electrically stimulated dental implants triggers soft-tissue integration and bactericidal functions. Adv. Funct. Mater. 34, 2311027. 10.1002/adfm.202311027

[B24] Jia B. Zhang B. Li J. Qin J. Huang Y. Huang M. (2024). Emerging polymeric materials for treatment of oral diseases: design strategy towards a unique oral environment. Chem. Soc. Rev. 53, 3273–3301. 10.1039/d3cs01039b 38507263

[B25] Jiang W. Deng Z. Dai X. Zhao W. (2021). PANoptosis: a new insight into oral infectious diseases. Front. Immunol. 12, 789610. 10.3389/fimmu.2021.789610 34970269 PMC8712492

[B26] Kabakov P. Kim T. Cheng Z. Jiang X. Zhang S. (2023). The versatility of piezoelectric composites. Annu. Rev. Mater. Res. 53, 165–193. 10.1146/annurev-matsci-080921-092839

[B27] Kapat K. Shubhra Q. T. H. Zhou M. Leeuwenburgh S. (2020). Piezoelectric nano-biomaterials for biomedicine and tissue regeneration. Adv. Funct. Mater. 30, 1909045. 10.1002/adfm.201909045

[B28] Kim D. Bae J. Heo J. H. Park C. H. Kim E. B. Lee J. H. (2022). Nanoparticles as next-generation tooth-whitening agents: progress and perspectives. ACS Nano 16 (7), 10042–10065. 10.1021/acsnano.2c01412 35704786

[B29] Kim D. Han S. A. Kim J. H. Lee J. Kim S. Lee S. (2020). Biomolecular piezoelectric materials: from amino acids to living tissues. Adv. Mater. 32, 1906989. 10.1002/adma.201906989 32103565

[B30] Lai D. Ma W. Wang J. Zhang L. Shi J. Lu C. (2023). Immune infiltration and diagnostic value of immune-related genes in periodontitis using bioinformatics analysis. J. Periodont. Res. 58, 369–380. 10.1111/jre.13097 36691896

[B31] Li J. Zhao X. Xia Y. Qi X. Jiang C. Xiao Y. (2024b). Strontium‐containing piezoelectric biofilm promotes dentin tissue regeneration. Adv. Mater. 36, 2313419. 10.1002/adma.202313419 38335452

[B32] Li P. Ou Q. Shi S. Shao C. (2023). Immunomodulatory properties of mesenchymal stem cells/dental stem cells and their therapeutic applications. Cell. Mol. Immunol. 20 (6), 558–569. 10.1038/s41423-023-00998-y 36973490 PMC10040934

[B33] Li X. Tian B. M. Deng D. K. Liu F. Zhou H. Kong D. Q. (2022). LncRNA GACAT2 binds with protein PKM1/2 to regulate cell mitochondrial function and cementogenesis in an inflammatory environment. Bone Res. 10 (1), 29. 10.1038/s41413-022-00197-x 35296649 PMC8927299

[B34] Li Y. Liu Y. Cui J. Zhu M. Wang W. Chen K. (2024a). Oral-gut microbial transmission promotes diabetic coronary heart disease. Cardiovasc. Diabetol. 23, 123. 10.1186/s12933-024-02217-y 38581039 PMC10998415

[B35] Liu Q. Liu L. Fan D. Xie S. Wang C. Gou X. (2024b). Self-powered biodegradable piezoelectric fibrous composites as antibacterial and wound healing dressings. Appl. Mater. Today 37, 102120. 10.1016/j.apmt.2024.102120

[B36] Liu X. Wan X. Sui B. Hu Q. Liu Z. Ding T. (2024a). Piezoelectric hydrogel for treatment of periodontitis through bioenergetic activation. Bioact. Mater. 35, 346–361. 10.1016/j.bioactmat.2024.02.011 38379699 PMC10876489

[B37] Lu M. Xuan S. Wang Z. (2019). Oral microbiota: a new view of body health. Food Sci. Hum. well. 8 (1), 8–15. 10.1016/j.fshw.2018.12.001

[B38] Luan J. Li R. Xu W. Sun H. Li Q. Wang D. (2023). Functional biomaterials for comprehensive periodontitis therapy. Acta Pharm. Sin. B 13 (6), 2310–2333. 10.1016/j.apsb.2022.10.026 37425066 PMC10326309

[B39] Luo S. Zhang C. Xiong W. Song Y. Wang Q. Zhang H. (2024). Advances in electroactive biomaterials: through the lens of electrical stimulation promoting bone regeneration strategy. J. Orthop. Transl. 47, 191–206. 10.1016/j.jot.2024.06.009 PMC11261049 39040489

[B40] Ma G. Wu A. Zhou S. Wang M. Zhang B. Liu Y. (2024). Tooth whitening and caries prevention toothbrush based on PTFE electret. J. Mater. Sci. 59, 2522–2533. 10.1007/s10853-024-09355-4

[B41] Mei H. Liu H. Sha C. Lv Q. Song Q. Jiang L. (2024). Multifunctional metal-phenolic composites promote efficient periodontitis treatment via antibacterial and osteogenic properties. ACS Appl. Mater. Interfaces 16 (11), 13573–13584. 10.1021/acsami.3c19621 38439708

[B42] Mi J. Zhi M. Kang W. Liang Q. Tang D. Wang T. (2024). Succession of the oral microbiome with the increasing severity of periodontitis. VIEW 5, 20230118. 10.1002/viw.20230118

[B43] Mokhtari F. Azimi B. Salehi M. Hashemikia S. Danti S. (2021). Recent advances of polymer-based piezoelectric composites for biomedical applications. J. Mech. Behav. Biomed. 122, 104669. 10.1016/j.jmbbm.2021.104669 34280866

[B44] Montoya C. Jain A. Londono J. J. Correa S. Lelkes P. I. Melo M. A. (2021a). Multifunctional dental composite with piezoelectric nanofillers for combined antibacterial and mineralization effects. ACS Appl. Mater. Interfaces 13, 43868–43879. 10.1021/acsami.1c06331 34494813

[B45] Montoya C. Kurylec J. Baraniya D. Tripathi A. Puri S. Orrego S. (2021b). Antifungal effect of piezoelectric charges on PMMA dentures. ACS Biomater. Sci. Eng. 7 (10), 4838–4846. 10.1021/acsbiomaterials.1c00926 34596379 PMC9228501

[B46] Moutsopoulos N. M. Konkel J. E. (2018). Tissue-specific immunity at the oral mucosal barrier. Trends Immunol. 39 (4), 276–287. 10.1016/j.it.2017.08.005 28923364 PMC5843496

[B47] Nain A. Chakraborty S. Barman S. R. Gavit P. Indrakumar S. Agrawal A. (2024). Progress in the development of piezoelectric biomaterials for tissue remodeling. Biomaterials 307, 122528. 10.1016/j.biomaterials.2024.122528 38522326

[B48] Pan Q. Zheng Y. Zhou Y. Zhang X. Yuan M. Guo J. (2024). Doping engineering of piezo-sonocatalytic nanocoating confer dental implants with enhanced antibacterial performances and osteogenic activity. Adv. Funct. Mater. 34, 2313553. 10.1002/adfm.202313553

[B49] Peng S. Fu H. Li R. Li H. Wang S. Li B. (2024). A new direction in periodontitis treatment: biomaterial-mediated macrophage immunotherapy. J. Nanobiotechnol. 359, 22. 10.1186/s12951-024-02592-4 PMC11193307 38907216

[B50] Peng X. Cheng L. You Y. Tang C. Ren B. Li Y. (2022). Oral microbiota in human systematic diseases. Int. J. Oral. Sci. 14, 14. 10.1038/s41368-022-00163-7 35236828 PMC8891310

[B51] Ren J. Wang X. Bao T. Shen X. Yin D. Liang Q. (2024). Piezoelectric dual network dressing with adaptive electrical stimulation for diabetic infected wound repair via antibacterial, antioxidant, anti-inflammation, and angiogenesis. Chem. Eng. J. 491, 151801. 10.1016/j.cej.2024.151801

[B52] Roldan L. Montoya C. Solanki V. Cai K. Q. Yang M. Correa S. (2023). A novel injectable piezoelectric hydrogel for periodontal disease treatment. ACS Appl. Mater. Interfaces 15 (37), 43441–43454. 10.1021/acsami.3c08336 37672788

[B53] Sedghi L. DiMassa V. Harrington A. Lynch S. V. Kapila Y. L. (2021). The oral microbiome: role of key organisms and complex networks in oral health and disease. Periodontol 87, 107–131. 10.1111/prd.12393 PMC8457218 34463991

[B54] Sharma A. Bhardwaj U. Jain D. Kushwaha H. S. (2022). NaNbO_3_/ZnO piezocatalyst for non-destructive tooth cleaning and antibacterial activity. iScience 25, 104915. 10.1016/j.isci.2022.104915 36060077 PMC9428797

[B55] Shi Y. Zhang N. Liu J. Wang J. Shen S. Zhang J. (2023). Preparation of nanocomposites for antibacterial orthodontic invisible appliance based on piezoelectric catalysis. Sensors 23, 5336. 10.3390/s23115336 37300063 PMC10256112

[B56] Smith M. Kar-Narayan S. (2022). Piezoelectric polymers: theory, challenges and opportunities. Int. Mater. Rev. 67, 65–88. 10.1080/09506608.2021.1915935

[B57] Song J. Lu Y. Pan T. Wang J. Liu Z. Xu L. (2024). Manipulation of surface electrical charge on nanocomposite membranes confers wide spectrum bactericidal effects and promotes tissue regeneration. Adv. Funct. Mater. 34 (22), 2314024. 10.1002/adfm.202314024

[B58] Tonelli A. Lumngwena E. N. Ntusi N. A. B. (2023). The oral microbiome in the pathophysiology of cardiovascular disease. Nat. Rev. Cardiol. 20, 386–403. 10.1038/s41569-022-00825-3 36624275

[B59] Wang J. Wu J. Zhang J. Guan L. Feng H. Zhu K. (2024b). Bibliometric and visualized analysis of piezoelectric materials in biomedical application. ACS Appl. Electron. Mater. 6 (3), 1562–1573. 10.1021/acsaelm.3c01596

[B60] Wang K. Han C. Li J. Qiu J. Sunarso J. Liu S. (2022a). The mechanism of piezocatalysis: energy band theory or screening charge effect? Angew. Chem. Int. Ed. 134 (6), e202110429. 10.1002/ange.202110429 34612568

[B61] Wang L. Zhang S. Zhang Y. An Q. (2023b). Piezodynamic therapy: mechanisms and biomedical applications. Nano Energy 110, 108342. 10.1016/j.nanoen.2023.108342

[B62] Wang X. Dai X. Chen Y. (2023c). Sonopiezoelectric nanomedicine and materdicine. Small 19, 2301693. 10.1002/smll.202301693 37093550

[B63] Wang X. Huan Y. Ji S. Zhu Y. Wei T. Cheng Z. (2022c). Ultra-high piezoelectric performance by rational tuning of heterovalent-ion doping in lead-free piezoelectric ceramics. Nano Energy 101, 107580. 10.1016/j.nanoen.2022.107580

[B64] Wang Y. Chang L. Gao H. Yu C. Gao Y. Peng Q. (2024a). Nanomaterials-based advanced systems for photothermal/photodynamic therapy of oral cancer. Eur. J. Med. Chem. 272 (5), 116508. 10.1016/j.ejmech.2024.116508 38761583

[B65] Wang Y. Wang S. Meng Y. Liu Z. Li D. Bai Y. (2022b). Pyro-catalysis for tooth whitening via oral temperature fluctuation. Nat. Commun. 13, 4419. 10.1038/s41467-022-32132-3 35906221 PMC9338087

[B66] Wang Y. Wen X. Jia Y. Huang M. Wang F. Zhang X. (2020). Piezo-catalysis for nondestructive tooth whitening. Nat. Commun. 11, 1328. 10.1038/s41467-020-15015-3 32165627 PMC7067860

[B67] Wang Y. Zang P. Yang D. Zhang R. Gai S. Yang P. (2023a). The fundamentals and applications of piezoelectric materials for tumor therapy: recent advances and outlook. Mater. Horiz. 10, 1140–1184. 10.1039/d2mh01221a 36729448

[B68] Wei Y. Hu X. Shao J. Wang S. Zhang Y. Xie W. (2024c). Daily sonic toothbrush triggered biocompatible BaTiO_3_/chitosan multiporous coating with enhanced piezocatalysis for intraoral antibacterial activity. Mater. Today Commun. 38, 107715. 10.1016/j.mtcomm.2023.107715

[B69] Wei Y. Liang Y. Qi K. Gu Z. Yan B. Xie H. (2024b). Exploring the application of piezoelectric ceramics in bone regeneration. J. Biomater. Appl., 8853282241274528. 10.1177/08853282241274528 39152927

[B70] Wei Y. Zheng L. Xie X. Yang X. Liao J. (2024a). Recent advances in stimuli responsive hydrogels for oral disease treatment. Mater. Des. 240, 112817. 10.1016/j.matdes.2024.112817

[B71] WHO (2022). Global oral health status report: towards universal health coverage for oral health by 2030. Geneva: World Health Organization.

[B72] Wu L. Gao H. Han Q. Guan W. Sun S. Zheng T. (2023). Piezoelectric materials for neuroregeneration: a review. Biomater. Sci. 11, 7296–7310. 10.1039/d3bm01111a 37812084

[B73] Xu H. Wang L. Zhao B. Zhao C. Lin K. (2023). Research and application progress of piezoelectric biomaterials in tissue regeneration. Prog. Biomed. Eng. 44, 1674–1242. 10.3969/j.issn.1674-1242.2023.02.001

[B74] Xu Q. Gao X. Zhao S. Liu Y. N. Zhang D. Zhou K. (2021). Construction of bio-piezoelectric platforms: from structures and synthesis to applications. Adv. Mater. 33, 2008452. 10.1002/adma.202008452 PMC11469329 34033180

[B75] Yang B. Pang X. Li Z. Chen Z. Wang Y. (2021). Immunomodulation in the treatment of periodontitis: progress and perspectives. Front. Immunol. 12, 781378. 10.3389/fimmu.2021.781378 34868054 PMC8640126

[B76] Zhang H. Tang Y. Gu Z. Wang P. Chen X. Lv H. (2024b). Biodegradable ferroelectric molecular crystal with large piezoelectric response. Science 383, 1492–1498. 10.1126/science.adj1946 38547269

[B77] Zhang L. Pan J. Zhang J. (2022). Integrated two-phase free radical hydrogel: safe, ultra-fast tooth whitening and antibacterial activity. J. Mater. Sci. Technol. 100, 59–66. 10.1016/j.jmst.2021.05.029

[B78] Zhang L. Yang T. Ding L. Chang M. Yin X. Chen Y. (2024c). Engineering 2D Bi_4_NbO_8_Br single crystalline nanosheets for piezoelectric and piezodynamic tumor nanotherapy. Chem. Eng. J. 484, 149445. 10.1016/j.cej.2024.149445

[B79] Zhang S. Zhang H. Sun J. Javanmardi N. Li T. Jin F. (2024a). A review of recent advances of piezoelectric poly-L-lactic acid for biomedical applications. Int. J. Biol. Macromol. 276, 133748. 10.1016/j.ijbiomac.2024.133748 38986996

[B80] Zhao R. Han W. Tang K. Shao R. Zhu P. Zhang S. (2022). Function of normal oral mucosa revealed by single-cell RNA sequencing. J. Cell. Biochem. 123, 1481–1494. 10.1002/jcb.30307 35894175

[B81] Zheng H. Lin H. Tian H. Lin K. Yang F. Zhang X. (2024). Steering piezocatalytic therapy for optimized tumoricidal effect. Adv. Funct. Mater. 34, 2400174. 10.1002/adfm.202400174

[B82] Zheng H. Zhou Y. Zheng Y. Liu G. (2023). Advances in hydrogels for the treatment of periodontitis. J. Mater. Chem. B 11, 7321–7333. 10.1039/d3tb00835e 37431231

[B83] Zhou W. Liang J. Huang X. Weir M. D. Masri R. Oates T. W. (2024). Novel antibacterial titanium implant healing abutment with dimethylaminohexadecyl methacrylate to combat implant-related infections. Dent. Mater. 40 (2), 244–253. 10.1016/j.dental.2023.11.011 37981511

